# Evaluation of Pancreatic Lesions: Comparative Analysis of Endoscopic Ultrasonography, Computed Tomography, and Magnetic Resonance Imaging

**DOI:** 10.3390/medicina62050982

**Published:** 2026-05-17

**Authors:** Seher Hekimsoy, Aliye Soylu, Mehmet Bayram, Hafize Uzun, Omur Tabak

**Affiliations:** 1 Department of Internal Medicine, Kanuni Sultan Suleyman Training and Research Hospital, University of Health Sciences, 34303 Istanbul, Türkiye; seherhekimsoy@hotmail.com; 2 Department of Gastroenterology, Bakirkoy Dr. Sadi Konuk Training and Research Hospital, University of Health Sciences, 34147 Istanbul, Türkiye; asoylu2@hotmail.com; 3 Department of Gastroenterology, Türkiye Hospital, 34365 Istanbul, Türkiye; drmhbayram@gmail.com; 4 Department of Medical Biochemistry, Faculty of Medicine, Istanbul Atlas University, 34403 Istanbul, Türkiye; huzun59@hotmail.com

**Keywords:** pancreatic lesion, endoscopic ultrasonography, computed tomography, magnetic resonance imaging

## Abstract

*Background and Objectives:* This study aimed to evaluate the diagnostic performance of endoscopic ultrasound (EUS) in comparison with computed tomography (CT) and magnetic resonance imaging (MRI) in the characterization of pancreatic lesions, and to assess the concordance of imaging findings with histopathological outcomes. *Materials and Methods:* A total of 76 patients who underwent EUS for pancreatic lesions between April 2021 and April 2022 were retrospectively analyzed. EUS findings were compared with CT and/or MRI in terms of lesion size, localization, and morphological characteristics. Histopathological results and laboratory parameters, including serum amylase, lipase, carcinoembryonic antigen (CEA), and carbohydrate antigen 19-9 (CA 19-9), were evaluated. Diagnostic performance metrics, including sensitivity, specificity, and accuracy, were calculated. *Results*: The study included 76 patients (55.3% male; mean age 59.93 ± 14.03 years). EUS demonstrated superior detection of lesions smaller than 2 cm (42.1% vs. 35.5%; *p* < 0.01) and a higher ability to identify solid components (17.1% vs. 9.7%; *p* < 0.01) compared to cross-sectional imaging. While overall sensitivity for malignancy was comparable between modalities, EUS showed higher specificity (58.33%) and diagnostic accuracy (55.26%). Pancreatic duct dilation, solid lesion morphology, larger lesion size, and elevated CA 19-9 levels were significantly associated with malignant pathology (*p* < 0.05). A descriptive agreement analysis revealed moderate concordance between EUS and radiological imaging in lesion size classification and morphological characterization. *Conclusions*: EUS demonstrates superior performance in detecting small pancreatic lesions and identifying solid components associated with malignancy. Although its sensitivity is comparable to CT and MRI, its higher specificity and diagnostic accuracy support its important role in lesion characterization. However, EUS should be considered complementary to cross-sectional imaging within a multimodal diagnostic approach. Integration of imaging findings with biochemical markers may further enhance diagnostic accuracy and clinical decision-making. Larger prospective studies with standardized protocols are warranted to validate these findings.

## 1. Introduction

Identifying pancreatic lesions requires an integrated approach combining clinical assessment, laboratory markers, and cross-sectional imaging. Current diagnostic standards generally rely on contrast-enhanced computed tomography (CT) and magnetic resonance imaging/magnetic resonance cholangiopancreatography (MRI/MRCP) as first-line modalities for lesion detection, anatomical assessment, ductal evaluation, and treatment planning [1,2]. While some pancreatic lesions present with symptoms, many are detected incidentally due to the increasing use of high-resolution abdominal imaging. Recent guidelines and meta-analyses emphasize the complementary roles of CT, MRI, and endoscopic ultrasound (EUS) in the evaluation of pancreatic lesions [1,2,3,4].

Pancreatic cystic lesions include both non-neoplastic and neoplastic entities, such as pseudocysts, serous cystic neoplasms, mucinous cystic neoplasms, intraductal papillary mucinous neoplasms, and solid pseudopapillary neoplasms [3]. The main clinical challenge is not only detecting these lesions but also differentiating benign lesions from premalignant or malignant lesions to avoid both delayed cancer diagnosis and unnecessary surgical intervention [1,2].

Despite advances in CT and MRI, definitive characterization of pancreatic lesions may remain difficult, particularly in small lesions, indeterminate cystic lesions, and lesions with subtle solid components [2]. EUS provides high-resolution visualization of the pancreas and enables tissue or cyst fluid sampling when indicated; however, its role should be considered complementary rather than universally superior to CT or MRI [4,5]. Recent evidence suggests that the diagnostic performance of CT, MRI, and EUS varies according to lesion type, size, morphology, operator expertise, and availability of tissue acquisition techniques [4,6].

Therefore, the incremental value of EUS is most clinically relevant in selected scenarios, including small lesions, indeterminate findings on cross-sectional imaging, suspected solid components, ductal communication, mural nodules, and cases requiring histopathological confirmation [1,4,5]. In this context, real-world data comparing EUS with CT/MRI and correlating imaging findings with histopathological outcomes may help clarify the practical role of multimodal imaging in routine clinical decision-making.

Accordingly, the present study aimed to compare EUS with CT and MRI in the evaluation of pancreatic lesions, focusing on lesion detection, size classification, morphological characterization, and concordance with histopathological outcomes. The study also aimed to assess clinically relevant indicators associated with malignancy, including ductal dilation, solid morphology, lesion size, and serum CA 19-9 levels. This study aims to provide real-world evidence on the comparative performance of imaging modalities in routine clinical practice, addressing a gap between controlled studies and daily clinical decision-making.

## 2. Materials and Methods

### 2.1. Ethical Considerations

Ethical approval for this study was obtained from the Institutional Review Board of the University of Health Sciences, Kanuni Sultan Süleyman Training and Research Hospital (Approval Date: 5 January 2022; Decision No: 2022.01.10). All procedures performed were in accordance with the 1964 Helsinki Declaration and its later amendments.

### 2.2. Study Design and Patient Selection

This retrospective study was conducted at the Department of Gastroenterology, University of Health Sciences, Kanuni Sultan Süleyman Training and Research Hospital. A total of 76 patients who underwent EUS for pancreatic lesions between April 2021 and April 2022 were included. Patient data, including EUS findings, histopathological results, cross-sectional imaging (CT and/or MRI), and laboratory parameters, were retrieved from the hospital’s electronic database.

Patients were evaluated in routine clinical practice, primarily due to suspected pancreatic pathology based on clinical symptoms, abnormal laboratory findings, or incidental imaging findings. None of the patients were part of a formal screening program.

The inclusion criteria were:

- Age ≥ 18 years.
- Availability of EUS and at least one cross-sectional imaging modality (CT and/or MRI).

The exclusion criteria were:

- Age < 18 years.
- Presence of active systemic infection or sepsis.
- Incomplete clinical, radiological, or laboratory data.
- Radiological examinations that were inconclusive or lacked sufficient diagnostic clarity.

Of the 76 cases, 33 underwent CT and 43 underwent MRI, with 20 patients having both imaging modalities available. In cases where multiple imaging studies were present, the examination performed closest to the date of the EUS procedure and providing the most clinically relevant diagnostic information was selected. The choice of imaging modality (CT vs. MRI) was based on clinical indication, availability, and physician preference, reflecting real-world clinical practice.

A descriptive subgroup analysis was performed for patients who underwent all three imaging modalities (EUS, CT, and MRI).

Biochemical markers (CA 19-9, CEA, amylase, and lipase) were evaluated to explore their association with malignancy and to complement imaging findings.

Follow-up data were retrospectively obtained from hospital records. Patients who underwent EUS between March 2021 and January 2022 were included in the study. The median follow-up duration was approximately 12 months, and follow-up information was available for all patients included in the analysis. Survival status was assessed based on all-cause mortality. Due to the retrospective design, cause-specific mortality could not be reliably determined.

Both cystic and solid lesions were included in the analysis to reflect real-world clinical practice.

### 2.3. EUS Procedure and Lesion Evaluation

All EUS procedures were performed after a minimum of 8 h of fasting. Patients were placed in the left lateral position and received intravenous sedation with propofol, fentanyl, and midazolam. A PENTAX EPK-i5000 processor (PENTAX Medical, Redwood City, CA, USA) and compatible echoendoscopes were used.

During EUS examination, lesion type (solid or cystic), anatomical location, maximal diameter, and morphological features (septation, lobulation, mural nodules, communication with the pancreatic duct, and ductal dilation) were systematically recorded. The presence of lymphadenopathy, vascular involvement, and adjacent organ invasion was also evaluated.

Imaging assessments were derived from routine clinical reports prepared by different radiologists and EUS practitioners. Due to the retrospective design, blind interpretation was not feasible.

### 2.4. EUS-Guided Fine-Needle Aspiration (EUS-FNA)

EUS-guided tissue acquisition was performed according to standard clinical practice at our center. Prior to EUS-guided aspiration, complete blood count and coagulation profiles were reviewed, and anticoagulant/antiplatelet therapies were managed according to current guidelines. Prophylactic ciprofloxacin was administered intravenously before the procedure and continued orally for three days post-procedure.

Aspiration was typically performed using a 22 G needle; however, a 19 G needle was preferred for large, thick-walled, or suspected mucinous cysts. The aspirated cyst fluid was primarily allocated for cytopathological examination. In cases where more than 1 cc of fluid was obtained, biochemical analysis (amylase, lipase, CEA, and CA 19-9) was performed.

The number of passes was determined at the discretion of the endoscopist based on lesion characteristics and adequacy of the obtained material. Due to the retrospective nature of the study, detailed information regarding specific sampling techniques, including fanning, wet versus dry suction, and the number of actuations, was not consistently available for all cases. Rapid on-site evaluation (ROSE) or macroscopic on-site evaluation (MOSE) was not routinely performed during the study period.

The procedure was conducted in accordance with current recommendations, including the ESGE guidelines and relevant randomized controlled trials [5,6].

Cytopathological results were categorized as benign, malignant, suspicious, or non-diagnostic. Final interpretation of EUS-FNA findings was made in conjunction with clinical, radiological, and follow-up data. In cases of non-diagnostic or inconclusive results, patient management was guided by repeat imaging, clinical follow-up, and multidisciplinary evaluation.

### 2.5. Histopathological Analysis

All cytological and pathological specimens were processed and evaluated by experienced pathologists at the Department of Pathology of the University of Health Sciences, Kanuni Sultan Süleyman Training and Research Hospital. Malignant lesions were defined based on histopathological confirmation. Benign lesions were classified either by histopathological confirmation or, in the absence of tissue diagnosis, by clinical and radiological follow-up demonstrating lesion stability (no increase in size, absence of suspicious morphological changes, and no development of secondary findings), based on expert consensus.

### 2.6. Statistical Analysis

Statistical analyses were performed using NCSS (Number Cruncher Statistical System) 2007 (Kaysville, UT, USA). Descriptive statistics were summarized as mean ± standard deviation (SD), median, frequency, percentage, and minimum–maximum values. The conformity of quantitative data to normal distribution was assessed using the Shapiro–Wilk test and graphical analyses. For comparisons of quantitative variables between two groups, Student’s *t*-test was used for normally distributed data, whereas the Mann–Whitney *U* test was applied for non-normally distributed data. Qualitative variables were compared using the Pearson chi-square test, Fisher–Freeman–Halton test, or Fisher’s exact test, as appropriate. The diagnostic performance of EUS and radiological imaging in predicting pathological outcomes was evaluated using sensitivity, specificity, positive predictive value (PPV), negative predictive value (NPV), and diagnostic accuracy analyses. Confidence intervals (95% CI) for diagnostic performance measures were calculated using Wilson’s binomial proportion method. In addition, concordance between EUS and cross-sectional imaging (CT/MRI) was assessed using descriptive agreement rates and Cohen’s kappa statistics based on lesion size classification (<2 cm vs. ≥2 cm), lesion morphology (cystic vs. solid), and lesion detection. Descriptive agreement rates were calculated using all patients with available comparative imaging data between EUS and CT/MRI. Cohen’s kappa analysis was performed only in patients with complete comparable categorical data for lesion size classification and lesion morphology. EUS was used as the reference modality for concordance analysis. Statistical significance was defined as a *p*-value < 0.05.

## 3. Results

### 3.1. Patient Characteristics and Clinical Data

This study was conducted between April 2021 and April 2022, involving a total of 76 patients. The cohort comprised 44.7% (*n* = 34) females and 55.3% (*n* = 42) males. The patients’ ages ranged from 23 to 91 years, with a mean age of 59.93 ± 14.03 years.

**Figure 1:**
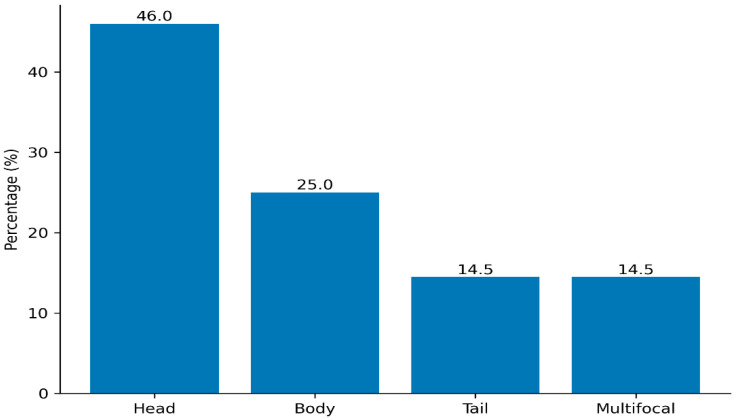
Distribution of lesion localization.

Regarding lesion localization, 46.0% (*n* = 35) were situated in the pancreatic head, 25.0% (*n* = 19) in the body, and 14.5% (*n* = 11) in the tail, while 14.5% (*n* = 11) exhibited multifocal involvement (Figure 1).

### 3.2. Pathological Outcomes and Survival Status

Pathological evaluation revealed malignancy in 21.1% (*n* = 16) of cases and benign findings in 78.9% (*n* = 60). Histopathological confirmation was available for all malignant lesions (*n* = 16). Among the 60 benign cases, 45 were histopathologically confirmed, while the remaining 15 were classified as benign based on clinical and radiological stability during follow-up (no increase in lesion size or development of suspicious features) and expert consensus. During the retrospective follow-up period, the survival rate was 85.5% (*n* = 65), whereas 14.5% (*n* = 11) of the patients were deceased. Pancreatic duct dilation was observed in 21.1% (*n* = 16) of the cases, and lymphadenopathy was present in 22.4% (*n* = 17) (Table 1).

Follow-up information was available for all patients included in the study. Patients who underwent EUS between March 2021 and January 2022 were retrospectively evaluated, with an approximate follow-up duration of 12 months based on hospital records. Survival status was assessed using all-cause mortality. A significantly higher mortality rate was observed in patients with malignant pathological outcomes compared to those with benign lesions.

### 3.3. Laboratory Findings

Serum Amylase, Lipase, CEA, and CA 19-9 levels were analyzed based on the institutional laboratory reference ranges (Amylase < 100 U/L, Lipase < 60 U/L, CEA 0–3.8 ng/mL, and CA 19-9 < 27 U/mL) (Table 2).

### 3.4. EUS and Radiological Imaging Findings

**Figure 2:**
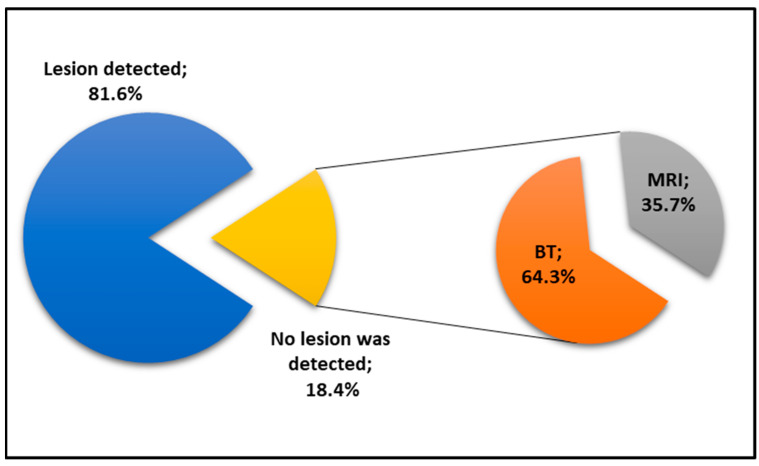
Distribution of lesion detection rates by computed tomography (CT) and magnetic resonance imaging (MRI).

Lesion sizes measured by EUS ranged from 5 to 130 mm, with a mean diameter of 28.91 ± 19.98 mm. Of these, 42.1% (*n* = 32) were smaller than 2 cm, and 57.9% (*n* = 44) were larger than 2 cm. Morphologically, 82.9% (*n* = 63) of the lesions were identified as cystic, while 17.1% (*n* = 13) were solid. Regarding radiological modalities, CT was performed in 43.4% (*n* = 33) of the patients, and MRI was performed in 56.6% (*n* = 43). Notably, radiological imaging failed to detect a lesion in 18.4% (*n* = 14) of the cases. Among these radiologically undetected lesions, 64.3% (*n* = 9) had been evaluated via CT and 35.7% (*n* = 5) via MRI (Figure 2).

The overall lesion size determined by combined radiological imaging (CT and MRI) ranged from 8 to 132.5 mm, with a mean diameter of 33.77 ± 22.22 mm. Among these, 35.5% (*n* = 22) were smaller than 2 cm, while 64.5% (*n* = 40) were larger than 2 cm. Morphologically, 90.3% (*n* = 56) of the lesions were cystic and 9.7% (*n* = 6) were solid.

### 3.5. CT Findings

Lesions were identified in 73.7% (*n* = 24) of patients undergoing CT. The mean size was 39.58 ± 18.57 mm (range: 14–132.5 mm), with 42.4% (*n* = 14) < 2 cm and 57.6% (*n* = 19) > 2 cm. Cystic and solid structures accounted for 87.5% (*n* = 21) and 12.5% (*n* = 3) of the lesions, respectively.

### 3.6. MRI Findings

Lesions were identified in 88.4% (*n* = 38) of patients who underwent MRI. The mean lesion size was 30.10 ± 18.57 mm (range, 8–80 mm). Lesions < 2 cm accounted for 51.2% (*n* = 22), whereas 48.8% (*n* = 21) were ≥2 cm. Many lesions were cystic (92.1%, *n* = 35), while only a small proportion were solid (7.9%, *n* = 3) (Table 3).

Among 32 lesions < 2 cm detected by EUS, 13 were not visualized on radiological imaging, while 19 were also identified as <2 cm. Of the 44 lesions ≥ 2 cm detected by EUS, 1 was not detected radiologically; 3 were misclassified as <2 cm, and 40 were correctly identified as ≥2 cm. A statistically significant difference was observed between EUS and radiological imaging (CT/MRI) in terms of lesion size (*p* = 0.001), with EUS demonstrating a higher detection rate for lesions < 2 cm.

Regarding lesion characteristics, among 63 lesions classified as cystic by EUS, 14 were not detected radiologically, while 49 were also identified as cystic. Of the 13 lesions classified as solid by EUS, 7 were misclassified as cystic and 6 as solid radiological imaging. A significant difference was found between EUS and radiological imaging in lesion characterization (*p* = 0.001), with EUS showing superior performance in detecting solid lesions.

Diagnostic performance parameters with corresponding 95% confidence intervals are presented in Table 4. For the detection of malignancy, overall radiological imaging demonstrated a sensitivity of 81.25%, specificity of 41.30%, positive predictive value (PPV) of 32.50%, negative predictive value (NPV) of 86.36%, and accuracy of 51.61%. CT showed a sensitivity of 85.71%, specificity of 23.53%, PPV of 31.57%, NPV of 80.00%, and accuracy of 41.67%, whereas MRI demonstrated a sensitivity of 77.78%, specificity of 51.72%, PPV of 33.33%, NPV of 88.24%, and accuracy of 57.89%. EUS yielded a sensitivity of 81.25%, specificity of 58.33%, PPV of 29.54%, NPV of 90.63%, and accuracy of 55.26%.

The mean age of patients with malignant lesions (69.81 ± 10.75 years; median: 71) was significantly higher than that of patients with benign lesions (57.30 ± 13.68 years; median: 58) (*p* < 0.001). However, no significant association was found between gender and pathological outcomes (*p* = 0.512), with malignancy rates of 17.6% (*n* = 6) in females and 23.8% (*n* = 10) in males.

### 3.7. Association of Clinicopathological and EUS Findings with Pathological Outcomes

The association between clinical, morphological, and biochemical parameters and pathological diagnosis is presented in Table 5. Ductal dilation was significantly more frequent in malignant cases compared to benign lesions (68.8% vs. 8.3%, *p* = 0.001). Similarly, solid lesion morphology was strongly associated with malignancy, whereas cystic lesions were predominantly benign (*p* = 0.001). Lesion size was also significantly associated with malignant pathology, with lesions ≥ 2 cm more frequently observed in malignant cases (29.5% vs. 9.4%, *p* = 0.044).

Among biochemical markers, lipase levels were significantly higher in malignant cases compared to benign ones (169.4 ± 219.6 vs. 53.8 ± 61.3, *p* = 0.026). In addition, both CA 19-9 and CEA levels were significantly elevated in malignant lesions (*p* = 0.001 for both), whereas amylase levels did not show a statistically significant association with malignancy (*p* = 0.136).

No significant differences were observed in lesion localization or the presence of lymphadenopathy between malignant and benign groups (*p* > 0.05). Mortality was significantly higher in patients with malignant lesions compared to those with benign pathology (62.5% vs. 1.7%, *p* = 0.001). Mortality data were based on all-cause mortality and should be interpreted with caution.

These parameters were analyzed to explore their association with malignancy and should be considered as supportive findings rather than primary outcomes of the study.

A descriptive agreement analysis between EUS and cross-sectional imaging demonstrated concordance rates of 77.6% for lesion size classification and 72.4% for morphological characterization. The overall lesion detection agreement was 81.6%. Discrepancies were primarily observed in lesions smaller than 2 cm and in the identification of solid components (Table 6).

**Figure 3:**
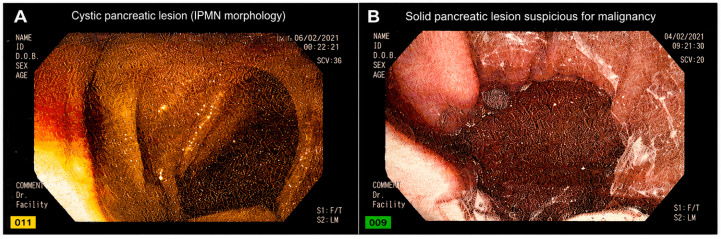
Representative endoscopic ultrasonography (EUS) images of pancreatic lesions. (**A**) Well-circumscribed cystic pancreatic lesion with septation features, consistent with IPMN morphology; (**B**) Heterogeneous hypoechoic solid pancreatic lesion with irregular margins suspicious for malignancy.

Representative EUS images of pancreatic lesions are presented in Figure 3. Figure 3A demonstrates a well-circumscribed cystic lesion with septation features consistent with IPMN morphology, whereas Figure 3B illustrates a heterogeneous hypoechoic solid lesion with irregular margins and necrotic components suspicious for malignancy. These representative images support the characteristic morphological findings identified during EUS evaluation.

Evaluation of EUS-FNA-related diagnostic interpretation demonstrated 8 true-positive, 60 true-negative, 1 false-positive, and 7 false-negative cases when compared with final pathological outcomes. The calculated sensitivity, specificity, and overall diagnostic accuracy were 53.3%, 98.4%, and 89.5%, respectively. The false-negative rate was 46.7%. Non-diagnostic or inconclusive cases were primarily related to insufficient material acquisition, necrotic sampling, or inability to safely perform tissue acquisition due to vascular proximity.

### 3.8. Agreement Between EUS and Cross-Sectional Imaging

Concordance analysis between EUS and cross-sectional imaging (CT/MRI) demonstrated a 93.5% agreement rate for lesion size classification (<2 cm vs. ≥2 cm), with substantial agreement observed on Cohen’s kappa analysis (κ = 0.762). Morphological characterization (cystic vs. solid) showed an agreement rate of 88.7%, corresponding to moderate agreement (κ = 0.537). Lesion detection agreement between modalities was 100% (Table 7). Agreement analyses were based on available paired imaging data between EUS and cross-sectional imaging modalities. Due to incomplete imaging availability across the cohort, the number of evaluable cases differed between descriptive agreement calculations and Cohen’s kappa analyses.

## 4. Discussion

This study evaluated the diagnostic efficacy of EUS and radiological imaging in differentiating malignant and benign pancreatic lesions, with a focus on clinically relevant morphological and biochemical predictors. Our findings demonstrate that advanced age, larger lesion size, and solid lesion morphology are significant indicators of malignancy. In addition, the presence of ductal dilatation and elevated biochemical markers, specifically lipase, CA 19-9, and CEA levels, was significantly associated with malignant pathological outcomes. However, these parameters were analyzed as supportive factors to complement imaging findings rather than as primary outcomes of the study. Furthermore, the observed association between malignancy and increased mortality rates underscores the importance of early and accurate differential diagnosis. Survival data were based on all-cause mortality and should therefore be interpreted with caution. While lesion localization and lymphadenopathy did not reach statistical significance, a trend toward higher malignancy rates in lesions located in the pancreatic head and in multifocal involvement was observed, providing additional clinical insight. Overall, these findings support the use of a multimodal, multiparametric approach integrating EUS-based morphological assessment with biochemical markers to improve risk stratification of pancreatic lesions.

The diagnosis and follow-up of pancreatic lesions have become increasingly feasible due to the widespread availability and use of high-quality cross-sectional imaging modalities. The evaluation of pancreatic lesions requires an integrated approach combining clinical findings, radiological imaging, EUS examination, and histopathological assessment. EUS offers several advantages, including high-resolution imaging and the ability to obtain tissue samples; however, it should be considered complementary to radiological imaging rather than a standalone modality.

In the present study, a total of 76 patients were analyzed retrospectively. The mean age of the cohort was 59.9 years. Patients with malignant pathology had a higher mean age (69.8 years) compared to those with benign lesions. This finding is consistent with previous reports indicating that pancreatic malignancies are more frequently diagnosed in older individuals, typically between 60 and 80 years of age, whereas occurrence in patients younger than 40 years is relatively uncommon [7,8].

When the gender distribution was examined, 55.3% of the patients were male and 44.7% were female. Previous studies have similarly reported a higher proportion of male patients, with rates of 62% and 52.1% [9,10]. Overall, the demographic profile of our cohort agreed with existing literature. Although gender was not significantly associated with pathological outcomes, the proportion of malignant cases was higher among male patients compared to females. This difference did not reach statistical significance, which may be attributed to the relatively small sample size.

In our study, lesions were most frequently detected in the pancreatic head, accounting for 46% of cases. This finding is consistent with previous reports; Cho et al. [11] reported that 63% of pancreatic cysts were in the pancreatic head, while Fernández-del Castillo et al. [12] observed a rate of 40%. Similarly, in our cohort, both pancreatic cancers and cystic lesions were most identified in the head of the pancreas [11,12].

Regarding lesion size, a statistically significant relationship was found between lesion diameter measured by EUS and malignancy. Lesions in the malignant group were larger (median diameter: 32.9 mm) compared to those in the benign group (27.8 mm). These findings are consistent with previous studies demonstrating that the risk of malignancy increases with lesion size, particularly in cysts exceeding 30 mm [13]. It has been reported that cysts measuring 3–5 cm carry an approximate malignancy risk of 15%, whereas this risk exceeds 30% when the diameter is greater than 5 cm [14]. When EUS and cross-sectional imaging (CT/MRI) were compared in terms of lesion size, EUS demonstrated a significantly higher detection rate for lesions smaller than 2 cm. This finding is clinically relevant, as smaller lesions may still harbor malignant potential and are more likely to be detected with high-resolution modalities such as EUS. In the study by Fernández-del Castillo et al. [12], approximately 20% of lesions measuring ≤2 cm was found to be malignant, with an additional proportion showing high-risk features, and these lesions could be more effectively identified with EUS. Overall, our results support the concept that lesion size is an important predictor of malignancy, while also highlighting the added value of EUS in detecting small yet clinically significant lesions.

When comparing EUS and radiological imaging in terms of lesion characterization, a statistically significant difference was observed, with EUS demonstrating a higher detection rate of solid components compared to CT/MRI. This finding is clinically relevant, as the presence of solid components is a well-recognized indicator of increased malignant potential. In addition, concordance analysis demonstrated substantial agreement between EUS and cross-sectional imaging for lesion size classification and moderate agreement for lesion morphology. Pais et al. [15] evaluated 74 patients with pancreatic cystic lesions and reported that EUS was more effective than radiological imaging in identifying solid components, which were considered a key feature associated with malignancy. Similarly, our findings align with previous studies demonstrating the added value of EUS in detecting solid components within pancreatic lesions [16,17,18]. However, rather than indicating absolute superiority, these results support the complementary role of EUS in conjunction with cross-sectional imaging, particularly in cases where detailed morphological assessment is required. Recent evidence and meta-analyses have demonstrated variability in diagnostic performance depending on lesion characteristics and clinical context [3,4].

In this study, the sensitivity, specificity, and diagnostic accuracy for malignancy were 81.25%, 41.30%, and 51.61% for radiological imaging overall; 85.71%, 23.53%, and 41.67% for CT; 77.78%, 51.72%, and 57.89% for MRI; and 81.25%, 58.33%, and 55.26% for EUS, respectively. These findings demonstrate considerable variability in diagnostic performance across imaging modalities, which is consistent with the heterogeneous results reported in the literature for pancreatic lesion characterization. In the study by Johnson et al. [19], the diagnostic accuracy of CT for malignant lesions was reported to be as high as 93–95%, whereas Curry et al. [20] reported substantially lower accuracy rates ranging from 23% to 41%. Similarly, Sperti et al. [21] reported a CT sensitivity of 65%, specificity of 88%, and diagnostic accuracy of 80% in differentiating malignant lesions. Such variability may be attributed to differences in study design, patient populations, lesion characteristics, and reference standards. In our study, the diagnostic performance of CT was closer to the lower range reported by Curry et al. [20], which may reflect real-world clinical conditions, including heterogeneous imaging quality and patient selection. In addition, biochemical and cytological parameters such as elevated serum CA 19-9, positive cytology, and increased cyst fluid markers may further aid in identifying lesions requiring surgical intervention; however, these should be interpreted as complementary to imaging findings rather than standalone decision criteria.

While the diagnostic accuracy of EUS was reported to be as high as 92–96% in the study by Koito et al. [22], it was found to be approximately 51% in the study by Brugge et al. [23]. In the study by Gerke et al. [24], the sensitivity of EUS in differentiating malignant lesions ranged from 48% to 87%, with specificity between 49% and 80% and diagnostic accuracy between 65% and 67%. Similarly, Sedleck et al. [25] reported a sensitivity of 91%, specificity of 60%, and diagnostic accuracy of 82% in distinguishing neoplastic from non-neoplastic lesions. In the present study, the sensitivity, specificity, and diagnostic accuracy of EUS were more comparable to the lower-to-moderate ranges reported by Brugge et al. and Gerke et al. [23,24]. These findings further support the considerable variability in EUS performance reported in the literature. Such variability is likely multifactorial and may be attributed to differences in operator experience, lesion characteristics, patient selection, and the availability of adjunctive techniques such as tissue acquisition. Therefore, the diagnostic performance of EUS should be interpreted within the context of the clinical setting and expertise, rather than as a fixed parameter.

In the present study, the presence of pancreatic ductal dilatation was found to be significantly higher in patients with malignancy compared to those without, which is consistent with previous reports. In a study by Pais et al. [15], involving 74 patients who underwent surgery for pancreatic cystic lesions, EUS examinations demonstrated that the presence of solid components, cyst wall thickening, and pancreatic ductal dilatation were key features associated with malignancy. In addition, a statistically significant association was observed between serum CA 19-9 levels and pathological outcomes. CA 19-9 levels were markedly higher in malignant cases (mean: 1647 U/mL) compared to benign cases (233 U/mL). Although there is no laboratory test that can be used as a standalone screening tool for pancreatic malignancy, CA 19-9 remains the most widely utilized serum biomarker in clinical practice. Previous studies have reported a sensitivity of approximately 80% and a specificity of 90%, with diagnostic performance influenced by tumor burden and lesion size [26]. However, CA 19-9 has important limitations, particularly in the detection of small or early-stage tumors, and may be elevated in benign conditions such as cholangitis or biliary obstruction [27]. In the study by Sperti et al. [21], evaluating serum tumor markers in 48 patients with pancreatic cystic lesions, CA 19-9 levels were found to be significantly higher in neoplastic cysts, supporting its role as a supportive diagnostic parameter.

In the present study, a statistically significant association was observed between mortality and pathological outcomes. Ten of the 16 patients with malignant pathology died during the follow-up period, indicating a substantially higher mortality rate in this group. The estimated survival rate during follow-up was approximately 37%; however, these findings should be interpreted with caution, as survival analysis was based on all-cause mortality and the follow-up duration was limited. Previous studies have shown that early-onset pancreatic cancer patients may present with distinct demographic and clinical characteristics, including male predominance and more advanced disease at diagnosis, while overall survival outcomes remain comparable to those of older pancreatic ductal adenocarcinoma patients [28]. Recent reports also indicate modest improvements in pancreatic cancer survival, although prognosis remains strongly stage-dependent, with significantly better outcomes in localized disease compared to metastatic disease [29]. In this context, the observed association between malignancy and increased mortality in our cohort further emphasizes the clinical importance of early and accurate characterization of pancreatic lesions.

Despite these strengths, several limitations should be acknowledged. The retrospective design introduces potential selection and information bias, while the relatively small sample size may limit statistical power and generalizability. In addition, heterogeneity in CT and MRI examinations performed at different centers and interpreted by different radiologists may have contributed to inter-observer variability. Imaging interpretations were based on routine clinical reports without standardized blinding procedures, which may have introduced interpretation bias and reflects real-world clinical practice. Detailed procedural data regarding EUS-guided tissue acquisition techniques (e.g., fanning, suction methods, and number of actuations) were not consistently available, and ROSE/MOSE was not routinely implemented. Moreover, EUS-FNA has inherent limitations, including false-negative and non-diagnostic results, particularly in lesions with necrotic components, technically challenging localization, or insufficient tissue acquisition. The relatively high false-negative rate observed in our cohort further highlights these limitations. Therefore, EUS-FNA findings should be interpreted in combination with imaging and clinical data. The absence of histopathological confirmation in all cases may also introduce verification bias and a risk of misclassification. In such instances, benign classification was based on clinical and radiological stability during follow-up, an approach consistent with previous studies on pancreatic cystic and indeterminate lesions [12], but one that should be interpreted with caution. In addition, cystic and solid lesions were analyzed together despite their distinct biological behavior, which may have influenced the overall assessment of diagnostic performance. Finally, biochemical markers and survival data were included as supportive rather than primary analyses, and mortality evaluation was limited to all-cause mortality because cause-specific data were not consistently available. Future prospective, multicenter studies with standardized imaging and tissue acquisition protocols are needed to validate these findings. Current guidelines further emphasize the importance of optimized sampling techniques and standardized procedural approaches to improve diagnostic yield in EUS-guided tissue acquisition [5,6].

In conclusion, our findings demonstrate that EUS provides enhanced performance in the detection of small pancreatic lesions and in the identification of solid components, both of which are critical indicators of malignancy. While the overall sensitivity of EUS and cross-sectional imaging modalities was comparable, the relatively higher specificity and diagnostic accuracy of EUS support its role in detailed lesion characterization. Importantly, these results do not suggest replacement of CT or MRI but rather highlight the complementary role of EUS within a multimodal diagnostic approach. The integration of EUS with cross-sectional imaging and biochemical markers may improve diagnostic confidence and clinical decision-making. These findings should be interpreted in the context of real-world clinical practice, where imaging selection and diagnostic pathways are not always standardized. Although the retrospective design and relatively small sample size represent inherent limitations, the overall consistency between imaging findings and histopathological outcomes supports the clinical relevance of our results. Future prospective, multicenter studies with standardized imaging and tissue acquisition protocols are needed to validate these findings and to further define the role of EUS in diagnostic algorithms for pancreatic lesions.

## Figures and Tables

**Table 1:** Distribution of morphological characteristics.

Variables	Category	*n*	%
Lesion Localization	Head	35	46.0
	Body	19	25.0
	Tail	11	14.5
	Multifocal	11	14.5
Ductal Dilation	Present	16	21.1
	Absent	60	78.9
Lymphadenopathy	Present	17	22.4
	Absent	59	77.6
Pathological Outcome	Malignant	16	21.1
	Benign	60	78.9
Final Status	Alive	65	85.5
	Deceased	11	14.5

**Table 2:** Distribution of laboratory characteristics.

Laboratory Findings	Statistics	Value	%
Amylase (U/L)	*Min-Max (Median)*	10–438 (65)	
	*Mean ± SD*	92.87 ± 90.31	
	Normal	60	78.9
	Anormal	16	21.1
Lipase (U/L)	*Min-Max (Median)*	6.6–793 (38)	
	*Mean ± SD*	78.11 ± 121.88	
	Normal	54	71.1
	Anormal	22	28.9
CA 19-9 (U/mL)	*Min-Max (Median)*	1.8–10,000 (12.1)	
	*Mean ± SD*	530.87 ± 1850.47	
	Normal	11	14.5
	Anormal	65	85.5
CEA (ng/mL)	*Min-Max (Median)*	0.3–42.9 (1.8)	
	*Mean ± SD*	3.21 ± 5.92	
	Normal	74	97.4
	Anormal	2	2.6

CA 19-9, Carbohydrate Antigen 19-9; CEA, Carcinoembryonic Antigen.

**Table 3:** Distribution of radiological findings based on CT and MRI.

		Total Radiology (CT + MRI) (*n* = 62)		CT (*n* = 33)		MRI (*n* = 43)	
		*n*	%	*n*	%	*n*	%
Imaging Modality	CT	33	43.4	33	100	-	-
	MRI	43	56.6	-	-	43	100
Lesion detection	Present	62	81.6	24	73.7	38	88.4
	Absent	14	18.4	9	27.3	5	11.6
Lesion size (mm)	*Min-Max (Median)*	8–132.5 (26.8)		14–132.5 (35.5)		8–80 (24.8)	
	*Mean ± SD*	33.77 ± 22.22		39.58 ± 18.57		30.10 ± 18.57	
	<2 cm	22	35.5	14	42.4	22	51.2
	>2 cm	40	64.5	19	57.6	21	48.8
Lesion morphology	Cystic	56	90.3	21	87.5	35	92.1
	Solid	6	9.7	3	12.5	3	7.9

CT, Computed Tomography; MRI, Magnetic Resonance Imaging.

**Table 4:** Diagnostic performance of imaging modalities in predicting malignancy.

Diagnostic Scan	Sensitivity % (95% CI)	Specificity % (95% CI)	Positive Predictive Value % (95% CI)	Negative Predictive Value % (95% CI)	Diagnostic Accuracy % (95% CI)
Radiological (*n* = 62)	81.25 (57.0–93.4)	41.30 (28.3–55.7)	32.50 (20.1–48.0)	86.36 (66.7–95.3)	51.61 (39.4–63.6)
CT (*n* = 24)	85.71 (48.7–97.4)	23.53 (9.6–47.3)	31.57 (15.4–54.0)	80.00 (37.6–96.4)	41.67 (24.5–61.2)
MRI (*n* = 38)	77.78 (45.3–93.7)	51.72 (34.4–68.6)	33.33 (17.2–54.6)	88.24 (65.7–96.7)	57.89 (42.2–72.1)
EUS (*n* = 76)	81.25 (57.0–93.4)	58.33 (46.0–69.7)	29.54 (18.2–44.2)	90.63 (75.8–96.8)	55.26 (44.1–65.9)

CT, Computed Tomography; MRI, Magnetic Resonance Imaging; EUS, Endoscopic Ultrasonography; CI, Confidence Interval. Confidence intervals (95% CI) were calculated using Wilson’s binomial method.

**Table 5:** Association of Clinical, Biochemical, and EUS Findings with Pathological Diagnosis.

		Pathological Findings		*p*
		Malignant	Benign	
Lesion Localization	Head	10 (28.6)	25 (71.4)	*^b^ 0.069*
	Body	2 (10.5)	17 (89.5)	
	Tail	0 (0)	11 (100)	
	Multifocal	4 (36.4)	7 (63.6)	
Ductal Dilation	Present	11 (68.8)	5 (31.3)	^d^ 0.001 **
	Absent	5 (8.3)	55 (91.7)	
Lymphadenopathy	Present	6 (35.3)	11 (64.7)	*^d^ 0.173*
	Absent	10 (16.9)	49 (83.1)	
Amylase (U/L)	*Min-Max (Median)*	10–408 (78)	22–438 (61)	*^e^ 0.136*
	*Mean ± SD*	145.13 ± 132.78	78.93 ± 70.33	
	Normal	9 (15.0)	51 (85.0)	
	Anormal	7 (43.8)	9 (56.3)	
Lipase (U/L)	*Min-Max (Median)*	8.5–793 (78.7)	6.6–333 (36.5)	^e^ 0.026 *
	*Mean ± SD*	169.40 ± 219.59	53.77 ± 61.33	
	Normal	7 (13.0)	47 (87.0)	
	Anormal	9 (40.9)	13 (59.1)	
CA 19-9 (U/mL)	*Min-Max (Median)*	1.8–10,000 (233.5)	1.8–10,000 (10)	^e^ 0.001 **
	*Mean ± SD*	1647 ± 2889	233 ± 1342	
	Normal	1 (9.1)	10 (90.9)	
	Anormal	15 (23.1)	50 (76.9)	
CEA (ng/mL)	*Min-Max (Median)*	0.9–42.9 (4.4)	0.3–5.6 (1.7)	^e^ 0.001 **
	*Mean ± SD*	8.11 ± 11.72	1.90 ± 1.19	
	Normal	14 (18.9)	60 (81.1)	
	Anormal	2 (100)	0 (0)	
EUS Lesion Size (mm)	*Min-Max (Median)*	15–60 (31.8)	5–130 (21.3)	^e^ 0.044 *
	*Mean ± SD*	32.94 ± 11.56	27.83 ± 21.64	
	<2 cm	3 (9.4)	29 (90.6)	
	>2 cm	13 (29.5)	31 (70.5)	
EUS Lesion Morphology	Cystic	3 (18.8)	60 (81.2)	^d^ 0.001 **
	Solid	13 (81.2)	0 (0)	
Mortality	Alive	6 (37.5)	59 (98.3)	^d^ 0.001 **
	Deceased	10 (62.5)	1 (1.7)	

^b^ Fisher–Freeman–Halton Test; ^d^ Fisher’s Exact Test; ^e^ Mann–Whitney U Test: * *p* < 0.05, ** *p* < 0.01. CA 19-9, Carbohydrate Antigen 19-9; CEA, Carcinoembryonic Antigen; EUS, Endoscopic Ultrasonography.

**Table 6:** Descriptive agreement between EUS and radiological imaging (CT/MRI).

Parameter	Agreement (%)	Discordance (%)
Lesion size (<2 cm vs. ≥2 cm)	77.6%	22.4%
Lesion morphology (cystic/solid)	72.4%	27.6%
Lesion detection	81.6%	18.4%

Agreement analyses were based on patients with available paired image data. Cohen’s kappa statistics were calculated only for cases with complete categorical data suitable for concordance analysis.

**Table 7:** Concordance analysis between EUS and cross-sectional imaging (CT/MRI).

Parameter Evaluated	Agreement Rate (%)	Cohen’s Kappa (κ)	Interpretation
Lesion size classification (<2 cm vs. ≥2 cm)	93.5%	0.762	Substantial agreement
Morphological characterization (cystic vs. solid)	88.7%	0.537	Moderate agreement
Lesion detection	100%	Not applicable *	Complete agreement

Agreement analyses were performed using comparative findings between EUS and available cross-sectional imaging modalities (CT and/or MRI). Cohen’s kappa statistics were calculated for lesion size classification and morphological characterization. Agreement analyses were based on patients with available paired image data. Cohen’s kappa statistics were calculated only for cases with complete categorical data suitable for concordance analysis. * Kappa analysis for lesion detection could not be calculated because all lesions identified by radiological imaging were also detected by EUS, resulting in complete agreement without variability.

## Data Availability

The data underlying this article are available in the article. If needed, please contact the corresponding author.
